# Native *Bacillus*-Based Probiotic Consortia Suppress *Vibrio parahaemolyticus* and Restructure Hatchery Water Microbiomes in Shrimp Larval Systems

**DOI:** 10.3390/pathogens15030287

**Published:** 2026-03-06

**Authors:** Betty Pazmiño-Gomez, Karen Rodas-Pazmiño, Rodrigo Pazmiño-Pérez, Tania Tapia-Guijarro, Wilman Balcazar-Quimi, Samuel Valle-Asan, Salma Salazar-Vera, Martin Villalva-Vera, Deily Ochoa-Fajardo, Edgar Rodas-Neira

**Affiliations:** 1 Faculty of Science and Engineering, Universidad Estatal de Milagro UNEMI, Km. 1 ½ Via Virgen de Fátima, Milagro 091050, Guayas, Ecuador; krodasp2@unemi.edu.ec (K.R.-P.); carolinatapiagiojarro@gmail.com (T.T.-G.); wbalcazarq@unemi.edu.ec (W.B.-Q.); svallea@unemi.edu.ec (S.V.-A.); ssalazarv2@unemi.edu.ec (S.S.-V.); mvillalvav3@unemi.edu.ec (M.V.-V.); deilyochoa72@gmail.com (D.O.-F.); erodasn@unemi.edu.ec (E.R.-N.); 2 Facultad de Ciencias Agrarias, Universidad Agraria del Ecuador, Av. 25 de Julio y Pio Jaramillo, Guayaquil 090107, Guayas, Ecuador; rjpazmino@uagraria.edu.ec

**Keywords:** *Bacillus* probiotics, *Vibrio parahaemolyticus*, shrimp aquaculture, microbiome restructuring, biological control

## Abstract

Shrimp aquaculture is constrained by opportunistic bacterial pathogens, particularly *Vibrio parahaemolyticus*, whose proliferation in hatchery systems is shaped by microbial community structure. We evaluated the antagonistic activity and microbiome effects of two native *Bacillus*-based probiotic consortia (CN5, RS3) applied alone or combined (MIX) in shrimp larval culture water over 30 days, relative to a no-probiotic control. Treatments were assessed using standardized in vitro inhibition assays, 16S rRNA gene (V3–V4) amplicon sequencing, functional inference, and integrative multivariate and structural modeling. All probiotic treatments showed consistently high antagonistic activity against *V. parahaemolyticus*, whereas the control showed no inhibition. Amplicon profiling indicated treatment-associated microbiome restructuring, with increased *Bacillus* dominance and reduced relative abundance of *Vibrio* spp. under probiotic conditions. Multivariate analyses separated probiotic and control groups, and PLS-SEM identified *Bacillus* dominance as a central driver of antagonistic activity mediated by inferred bioactive functional potential, while water-quality variables had limited direct effects. Probiotics were administered directly to the culture water once daily after routine water exchange to 1 × 10^6^ CFU mL^−1^ (CN5 or RS3); MIX was applied 1:1 (*v*/*v*) at the same total dose.

## 1. Introduction

Shrimp aquaculture has expanded rapidly over the last two decades, yet recurrent disease outbreaks driven by opportunistic bacteria continue to constrain productivity, profitability, and sustainability. Among the most consequential threats is acute hepatopancreatic necrosis disease (AHPND). It has been tightly linked to *Vibrio parahaemolyticus* strains that became virulent after acquiring a plasmid encoding PirAB-like toxins, which can trigger acute mortality and severe hepatopancreatic lesions in penaeid shrimp [1,2,3,4,5]. Beyond PirAB-mediated pathology, AHPND-causing strains may also maintain antibacterial type VI secretion systems with diverse effector repertoires, enhancing ecological competitiveness within dense aquaculture microbiomes [6]. Whole-genome sequencing has further clarified geographic origins and dissemination of outbreak-associated lineages, reinforcing the dynamic evolution and mobility of virulence determinants in shrimp production systems [7]. Importantly, experimental evidence indicates that PirABVP toxins can aggravate broader vibriosis contexts, emphasizing that management must address both pathogen abundance and virulence potential across the culture cycle [8,9].

*Vibrios* are naturally abundant in coastal and marine systems and exhibit substantial genetic and ecological diversity, which makes hatchery water and larval reservoirs interfaces where background *Vibrio* populations can expand under intensive husbandry, feed inputs, and fluctuating physicochemical conditions. Recent reviews describe the biodiversity of *Vibrios* across ecological niches and the drivers of their emergence in aquatic food webs [10]. For *V. parahaemolyticus* specifically, pathogenesis is multifactorial and depends on host susceptibility, environmental conditions, and the accessory genome, which carries a range of fitness determinants and virulence factors beyond Pir toxins [11]. Consequently, disease mitigation strategies must target not only the presence of *Vibrio* spp. but also the ecological context that enables pathogenic dominance, persistence, and expression of virulence determinants.

Sustainable disease control in aquaculture increasingly prioritizes preventive approaches that reinforce microbial and environmental barriers against opportunistic pathogens. Probiotic bacteria are among the most widely investigated tools, acting through competitive exclusion, production of antimicrobials, nutrient competition, and system-level stabilization of rearing conditions. Early conceptual models positioned probiotics as biological control agents in aquaculture and highlighted their potential to reduce disease pressure while supporting host performance [12]. At a conceptual level, the effectiveness of probiotics is rarely attributable to a single mechanism; rather, it emerges from shifts in community interactions and resource partitioning that favor beneficial guilds and suppress pathogen success. Comparative sequence-based approaches have long been used to differentiate microbial pathogens across hosts and settings [13], and analogous principles underpin culture-independent profiling in aquaculture: probiotic establishment, pathogen suppression, and functional transitions are best evaluated at the level of whole communities rather than individual isolates alone.

Among candidate probiotics, *Bacillus* spp. are attractive because spores tolerate stressful rearing conditions and industrial handling, while vegetative cells can produce a spectrum of antimicrobial metabolites and enzymes that influence both pathogens and water quality. Reviews emphasize that reliable probiotics require systematic screening that includes robust identification, safety assessment, and mechanistic evaluation under relevant environmental conditions [14,15,16]. In shrimp systems, *Bacillus subtilis* supplementation has been associated with improved growth performance, enhanced digestive enzyme activity, modulation of immune gene expression, and increased resistance under disease challenge [17]. Administering *Bacillus* strains directly in rearing water has also been reported to enhance water quality and increase resistance against *Vibrio* infection, supporting the idea that probiotics can reshape the rearing environment as well as the host–microbe interface [18]. Broader syntheses further highlight the growing evidence base for *Bacillus* as probiotics in aquaculture and the mechanistic versatility of this genus [19]. At the molecular level, *Bacillus* lipopeptides represent a key class of bioactive compounds that can inhibit competitors and shape interbacterial interactions, offering a plausible functional bridge between *Bacillus* dominance and anti-*Vibrio* effects in mixed communities [20]. Complementary management strategies, such as biofloc technology, likewise rely on microbial community engineering to improve water quality and reduce pathogen pressure, reinforcing the central role of microbiome structure in disease-resilient production [21].

Despite strong rationale and extensive research, translating probiotics into consistent field performance remains challenging because efficacy depends on strain selection, dosing, formulation, and environmental context. In practice, native consortia derived from the local production environment may offer advantages in ecological compatibility and persistence, but their deployment requires rigorous validation that connects culture-based antagonism phenotypes with community-wide outcomes. Standardized in vitro approaches for antimicrobial activity assessment provide essential first-line evidence of antagonism and help triage candidates prior to in vivo challenge trials or farm-scale implementation [22]. However, culture-based assays alone cannot resolve the non-culturable fraction of hatchery microbiomes, nor can they quantify how probiotics restructure broader bacterial networks that influence pathogen success and functional potential.

High-throughput 16S rRNA amplicon sequencing enables community-scale evaluation of how hatchery interventions reshape microbial assemblages, provided that analyses follow reproducible pipelines, rigorous preprocessing, and curated reference resources [23,24,25,26,27,28,29,30,31]. Functional inference and integrative multivariate modeling can then link treatment-associated taxonomic shifts to predicted bioactive potential and antagonistic phenotypes, offering a mechanism-oriented framework for evaluating probiotic consortia when shotgun metagenomics and targeted metabolomics are not available [32,33,34,35,36,37,38,39,40,41,42,43,44,45,46].

From an ecological standpoint, pathogen control in shrimp hatcheries is best viewed as management of a dynamic microbial meta-community rather than suppression of a single taxon. Virulence plasmids and toxin repertoires explain why specific *V. parahaemolyticus* lineages can trigger AHPND [1,2,3,4,5], yet disease expression is shaped by background community competition, resource availability, and physicochemical stressors that modulate host susceptibility and microbial growth rates [6,7,8,9,10,11]. Accordingly, interventions that shift the community toward stable, competitive, and functionally protective states may reduce the probability that toxigenic vibrios reach critical abundances or express virulence determinants. Within this logic, *Bacillus*-based probiotics remain among the most practical tools for community-level engineering because spores withstand storage and delivery constraints, and *Bacillus* metabolism can contribute both direct antagonism and indirect system stabilization [14,15,16,17,18,19,20,21]. Nevertheless, variability across farms persists, often reflecting mismatches between probiotic strains and local conditions—an argument for prioritizing native consortia that are already adapted to the production environment.

Objective of this study. In this context, the present study aimed to evaluate native probiotic consortia (CN5 and RS3), individually and as a mixed consortium (MIX), for their capacity to suppress *V. parahaemolyticus* and restructure hatchery water microbiomes across a 30-day time course, relative to a no-probiotic control (CTRL). Specifically, we combined (i) standardized in vitro antagonism screening to quantify anti-*Vibrio* activity [22], (ii) 16S rRNA gene (V3–V4) amplicon profiling with reproducible pipelines to characterize taxonomic and diversity shifts [23,24,25,26,27,28,29,30,31], (iii) functional inference to estimate bioactive potential relevant to pathogen suppression [32,33,34,35], and (iv) integrative multivariate and structural modeling (PCA and PLS-SEM) to test mechanistic relationships linking water quality, microbial diversity, *Bacillus* dominance, *Vibrio* presence, inferred bioactive function, and antagonistic outcomes [36,38,39,40,41,42,43,44,45,46]. By integrating phenotype, community composition, inferred function, and mechanistic modeling, this work seeks to provide a high-resolution, field-relevant evaluation of native probiotic consortia as scalable biocontrol tools for disease-resilient shrimp hatchery management.

## 2. Materials and Methods

### 2.1. Experimental Design

An exploratory experimental design was implemented to (i) characterize bacterial community dynamics in *Penaeus vannamei* larval culture water and (ii) quantify the antagonistic potential of native *Bacillus*-based probiotic consortia against *Vibrio parahaemolyticus* by integrating culture-based screening, 16S rRNA gene sequencing (V3–V4), and statistical modeling. *Vibrio parahaemolyticus* was selected as the target pathogen due to its relevance in shrimp hatcheries and its established association with AHPND in affected production systems [1,2,3,4,5]. Additional genomic and ecological evidence supporting AHPND/*Vibrio* epidemiology and strain diversification was considered when defining the analytical scope [6,7,8,9,10,11].

Water was sampled from a larval reservoir located in Guayas Province (Ecuador) across four sampling moments separated by 10-day intervals (Day 0, 10, 20, and 30). At each time point, four experimental conditions were evaluated: two native probiotic consortia (CN5, RS3), a mixed consortium (MIX = CN5 + RS3), and a no-probiotic control (CTRL). Each treatment–time combination included eight independent biological replicates (4 treatments × 4 times × 8 replicates), yielding a total of 128 observations for SEM-aligned indices and downstream multivariate analyses (Table 1).

#### 2.1.1. Daily Hatchery Management During the 30-Day Cycle

Larval rearing units were operated under routine hatchery conditions during Days 0–30. Water exchange was performed at 10% of tank volume once daily during Days 0–10, increased to 20% once daily during Days 10–20, and 30% once daily during Days 20–30, using pre-aerated seawater adjusted to the target salinity. Aeration was provided continuously using air stones supplied by a centralized blower line, and tank hygiene included daily siphoning of settled organic debris and biofilm removal from tank walls. Larvae were stocked at 100 larvae L^−1^ at Day 0; density adjustments were performed at Days 10 and 20 by splitting cohorts into identical units to maintain comparable biomass and to avoid overcrowding. Feeding followed a stage-adapted hatchery schedule consisting of microalgae during early larval stages and Artemia nauplii plus a commercial microencapsulated larval diet during later stages, administered four times per day with rations adjusted to observed consumption. These husbandry practices were kept identical across treatments; only probiotic administration differed among groups.

#### 2.1.2. Probiotic Preparation and Administration

CN5 and RS3 were prepared as spore-enriched cell suspensions and standardized to a working concentration of 1 × 10^9^ CFU mL^−1^ based on plate counts on Tryptic Soy Agar (BD Difco, Franklin Lakes, NJ, USA). Treatments were administered directly to the larval culture water to achieve a final concentration of 1 × 10^6^ CFU mL^−1^ for CN5 and RS3. The mixed treatment (MIX) was prepared by combining CN5 and RS3 at equal proportions (1:1, *v*/*v*) prior to application to achieve the same final total probiotic concentration (1 × 10^6^ CFU mL^−1^ total). Probiotics were applied once daily immediately after routine water exchange throughout the 30-day period. CTRL units received the same carrier volume (sterile 0.85% saline) without probiotics to control for handling effects.

Naming conventions are as follows. CN5 and RS3 denote two native *Bacillus*-enriched probiotic consortia isolated from the production environment; MIX denotes the 1:1 (*v*/*v*) combination of CN5 and RS3; and CTRL denotes the no-probiotic control. For derived indices, “*Vibrio* presence” is used as shorthand for genus-level relative abundance (RA) of *Vibrio* (a quantitative proxy, not a binary detection metric), whereas “*Bacillus* dominance” denotes RA of *Bacillus* as a proxy for taxon dominance in the community.

**Figure 1:**
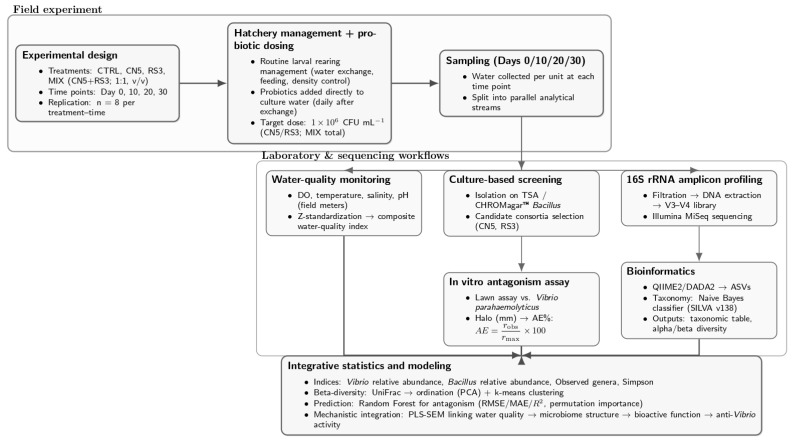
Experimental and analytical workflow of the study. The field experiment comprised four treatments (CTRL, CN5, RS3, and MIX [CN5 + RS3; 1:1, *v*/*v*]) evaluated across Days 0, 10, 20, and 30 with independent replication (*n* = 8 per treatment–time).

A schematic overview of the experimental and analytical workflow is provided in Figure 1, summarizing the field design (treatments, time points, replication, and probiotic dosing), the parallel laboratory streams (water-quality monitoring, culture-based screening and in vitro antagonism, and 16S rRNA amplicon profiling with subsequent bioinformatics), and the integrative statistical and mechanistic modeling used to link microbiome restructuring with anti-*Vibrio* activity.

### 2.2. Evaluated Variables

Six core response domains were evaluated and harmonized as SEM-aligned indices for integrative analysis shown in the following list and Table 2:

- **Anti-*Vibrio* activity**: inhibition halo diameter (mm) from a simultaneous inhibition/competitive exclusion assay; summarized as mean halo per observation. Antimicrobial evaluation procedures followed standardized in vitro guidance [22].
- ***Vibrio* presence (*Vibrio* relative abundance RA)**: genus-level relative abundance (RA) derived from taxonomic profiles, computed as the proportion of reads assigned to *Vibrio* spp. relative to total reads per sample ($n_{Vibrio}/N_{total}$), including *V. parahaemolyticus* when detected [10,11].
- ***Bacillus* dominance (*Bacillus* relative abundance RA; dominance proxy):** genus-level relative abundance (RA) of *Bacillus* spp. ($n_{Bacillus}/N_{total}$), interpreted as a dominance proxy under probiotic-enriched regimes [17,18,19].
- **Microbial diversity**: alpha-diversity index computed from ASV/feature tables (e.g., observed genera and Simpson diversity), supporting ecological interpretation of community restructuring.
- **Bioactive function**: index based on inferred functional potential using PICRUSt2 [32], interpreted with KEGG pathway hierarchies [33] and complementary orthology resources (eggNOG, COG) [34,35].

**Water quality**: For the composite water-quality index, each parameter was z-standardized across observations $\left(z_{x}=(x-\mu )/\sigma \right)$ and the index was computed as the mean of the z-scores for DO, salinity, temperature, and pH. The composite water-quality index (WQI) is a standardized score (mean z-scores) summarizing relative shifts in DO, salinity, temperature, and pH across the study observations as show in Equation (1); positive values indicate above-average conditions within the observed dataset, not necessarily an a priori ‘optimal’ state.

(1) $$WQI=\left(\frac{z_{DO}+z_{Sal}+z_{temp}+z_{pH}}{4}\right)\;$$

#### Water-Quality Instrumentation

Dissolved oxygen (DO; mg L^−1^) and temperature (°C) were measured in situ using a handheld multiparameter meter (ProDSS, YSI Inc., Yellow Springs, OH, USA). Salinity (ppt) was measured using a pocket digital refractometer (PAL series, ATAGO Co., Ltd., Tokyo, Japan), and pH was measured using a portable pH meter (HI98191, Hanna Instruments, Woonsocket, RI, USA). Instruments were calibrated according to manufacturers’ instructions prior to each sampling event.

Index calculations and interpretation. Genus-level relative abundance was computed as ${RA}_{g}$ = $n_{g}$/N, where $n_{g}$ is reads assigned to genus g and N is total reads per sample after quality control and ASV table construction. Richness (presence across taxa) was represented by Observed genera (number of genera with ${RA}_{g}$ > 0), whereas dominance/evenness was represented by the Simpson index (1 − Σ$p_{i}^{2}$), where $p_{i}$ is the relative abundance of genus *i*. Accordingly, *Vibrio* and *Bacillus* indices quantify treatment-associated changes in relative abundance, while alpha-diversity indices capture richness and dominance structure. Here, “presence” refers to genus-level relative abundance (a quantitative proxy for prevalence/expansion), not a binary presence/absence metric.

### 2.3. Biological Material

The biological material comprised:

1. culture water from penaeid shrimp larval production systems (matrix for microbiome profiling and probiotic screening);
2. two native probiotic consortia (CN5 and RS3) assembled from *Bacillus*-enriched isolates obtained from the same production environment, in line with probiotic selection principles in aquaculture [12,14,15,16];
3. a mixed consortium treatment (MIX) prepared by combining CN5 and RS3 at equal proportions; and
4. a no-probiotic control (CTRL).

*Vibrio parahaemolyticus* served as the target pathogen for antagonism assays because AHPND-linked virulence is associated with toxin-encoding plasmids (Pir-like toxins) and related genomic features [1,2,3,4,5,8,9].

### 2.4. Culture-Based Isolation and Morphotypic Screening

Culture-based screening used Tryptic Soy Agar (BD Difco, Franklin Lakes, NJ, USA) and chromogenic media for *Bacillus* differentiation (CHROMagar™ *Bacillus*, CHROMagar, La Plaine Saint-Denis, France). Plating on TSA and CHROMagar™ *Bacillus* (CHROMagar, La Plaine Saint-Denis, France) was used to enrich and differentiate *Bacillus*-like colonies for subsequent screening. Plates were incubated aerobically until visible colony development. Distinct colonies were selected based on morphology (size, texture, pigmentation, margin, elevation), re-streaked to purity, and preserved (TSA slants and/or glycerol stocks at −80 °C).

Two candidate consortia (CN5 and RS3) were defined as distinct pools of *Bacillus*-enriched isolates that showed consistent morphotypic profiles and inhibitory activity in preliminary screens, consistent with recommended screening steps for probiotic selection (Table 3) [14,15,16]. Mechanistically, *Bacillus*-associated antagonism was supported conceptually by lipopeptide-mediated inhibition and competitive interactions [20].

### 2.5. In Vitro Antagonism Assay and Antagonistic Effectiveness

Antagonistic activity against *Vibrio parahaemolyticus* was quantified using a simultaneous inhibition (competitive exclusion) assay on 90 mm Petri dishes. The target strain was a reference culture (*Vibrio parahaemolyticus*, ATCC 17802; American Type Culture Collection, Manassas, VA, USA) maintained at −80 °C in glycerol stocks.

For each assay, the strain was revived on TSA supplemented with 2% (*w*/*v*) NaCl (BD Difco, Franklin Lakes, NJ, USA) and grown in TSB + 2% NaCl at 30 °C for 16–18 h with agitation. The inoculum was standardized to ~1 × 10^8^ CFU mL^−1^ (OD600 ≈ 0.5), and 100 µL was spread uniformly onto TSA + 2% NaCl to form a confluent lawn. Consortia (CN5, RS3, MIX) were spotted at predefined positions, plates were incubated aerobically at 30 °C for 24 h, and inhibition halos were measured (mm) along two orthogonal axes and averaged per plate.

(2) $$AE\left(\%\right)=\left(\frac{r_{\mathrm{obs}}-r_{min}}{r_{\max}-r_{min}}\right)\times 100$$

where $r_{\mathrm{obs}}=D_{\mathrm{halo}}/2$ is the observed inhibition radius (mm) derived from the measured halo diameter $D_{\mathrm{halo}}$, and $r_{\max}=R_{\mathrm{plate}}-r_{\mathrm{inoc}}$ is the maximum measurable radius permitted by plate geometry ($R_{\mathrm{plate}}$ = 45 mm for 90 mm dishes; $r_{\min}$ ≈ 5 mm). Each treatment–time group included eight replicate plates.

### 2.6. Data Analysis

#### 2.6.1. DNA Extraction, Library Preparation, and Sequencing

For microbiome profiling, each water sample replicate was homogenized and an aliquot (250 mL) was filtered through a 0.45 µm membrane using vacuum filtration. DNA was extracted from membranes using DNeasy PowerWater Kit (QIAGEN, Hilden, Germany) following the manufacturer’s protocol. DNA quality/quantity were evaluated via NanoDrop™ 2000 spectrophotometer (Thermo Fisher Scientific, Waltham, MA, USA) and Qubit^®^ 4.0 fluorometer (Thermo Fisher Scientific, Waltham, MA, USA)., and integrity was checked by 1% agarose gel electrophoresis. The V3–V4 region of the 16S rRNA gene was amplified using universal primers 341F/805R, consistent with common primer evaluation for bacterial diversity studies [29]. Amplicons were purified, indexed, pooled, and sequenced on an Illumina MiSeq platform (Illumina, San Diego, CA, USA) (2 × 300 bp).

#### 2.6.2. Bioinformatic Processing and Taxonomic Assignment

Raw reads were processed in QIIME2 using DADA2 for denoising, paired-end merging, and chimera removal to generate ASVs [23,24]. Adapter removal and quality trimming followed standard preprocessing tools (Cutadapt and Trimmomatic) [25,26]. VSEARCH was used only for auxiliary processing steps (e.g., dereplication/support operations) and was not used for taxonomic assignment [27]. Accordingly, the QIIME 2 classify-consensus-vsearch taxonomy workflow was not used. Taxonomy was assigned using the Naïve Bayes classifier trained on SILVA (release 138) for the V3–V4 region [28]. Outputs (ASV table, taxonomy, metadata) were imported into R for downstream ecological processing with phyloseq [30]. Differential abundance testing across treatments and time points used DESeq2 with multiple-testing correction (Table 4) [31].

#### 2.6.3. Functional Inference and Annotation

Functional potential was inferred from ASVs using PICRUSt2 [32]. Predicted pathways were summarized against KEGG hierarchies [33] and complemented with orthology resources (eggNOG and COG) to support functional interpretation [34,35]. A “bioactive function” index was constructed by aggregating inferred functions plausibly related to antimicrobial biosynthesis, stress response, and competitive fitness.

#### 2.6.4. Multivariate Statistics (PCA)

Beta-diversity was computed using UniFrac distances [36]. For integrative visualization, PCA was performed on centered and scaled SEM-aligned indices using standard PCA definitions [38,39] and implemented in R via FactoMineR [40]. Cluster structure in PCA space was explored using k-means on leading PCs to summarize multivariate regimes.

#### 2.6.5. Machine Learning (Random Forest) for Out-of-Sample Validation

To quantify predictive generalization beyond in-sample modeling, a Random Forest regressor was trained to predict anti-*Vibrio* activity (halo mean, mm) from SEM-aligned indices (e.g., *Bacillus* dominance, *Vibrio* presence, microbial diversity, bioactive function, and water quality). Data were split into training and test partitions (e.g., 70/30), preserving treatment and time representation. Hyperparameters (number of trees, mtry/max_features, node size/min_samples_leaf) were tuned via cross-validation on the training set. Predictive performance on the test set was summarized using RMSE, MAE, and R^2^. Model interpretation used permutation importance to rank predictors by their contribution to predictive accuracy (Table 5).

#### 2.6.6. PLS-SEM Specification and Quality Assessment

A variance-based SEM (PLS-SEM) was specified to test a mechanistic cascade in which water quality and microbial diversity influence *Bacillus* dominance and *Vibrio* presence, which in turn shape inferred bioactive function and anti-*Vibrio* activity. Latent constructs were modeled reflectively with observed indicators (e.g., microbial diversity: observed genera and Simpson; water quality: dissolved oxygen, salinity, temperature, pH; anti-*Vibrio* activity: halo measurements; bioactive function: inferred KEGG/functional indicators). Reliability was assessed using Cronbach’s alpha [41]. Convergent validity used AVE and discriminant validity used Fornell–Larcker and HTMT [42,43]. Structural paths were estimated by non-parametric bootstrapping (e.g., 5000 resamples) and reported with R^2^, effect sizes (f^2^), and predictive relevance measures, following PLS-SEM guidance [44,45,46].

### 2.7. Null and Working Hypotheses

**H0-1 (treatment effect).**  *No differences exist among treatments (CN5, RS3, MIX, CTRL) in anti-Vibrio activity (mean inhibition halo) at any time point (0, 10, 20, 30).*

**H1-1.**  *At least one probiotic treatment (CN5, RS3, MIX) increases anti-Vibrio activity relative to CTRL, potentially varying with time.*

**H0-2 (community restructuring).**  *Treatments do not alter Vibrio presence, Bacillus dominance, microbial diversity, bioactive function, or the water-quality index across time.*

**H1-2.**  *Probiotic treatments reduce Vibrio presence and increase Bacillus dominance, with concomitant shifts in diversity, inferred functions, and/or water quality.*

**H0-3 (PCA separation).**  *Multivariate profiles (PCA scores) do not differ among treatments.*

**H1-3.**  *Treatments yield reproducible multivariate separation in PCA space consistent with distinct ecological regimes.*

**H0-4 (SEM paths).**  *All PLS-SEM structural path coefficients equal zero.*

**H1-4.**  *At least one SEM path is non-zero, consistent with a cascade where water quality/diversity affect Bacillus/Vibrio, which mediate bioactive function and antagonism.*

**H0-5 (Random Forest predictability).**  *SEM-aligned indices do not predict anti-Vibrio activity better than chance in held-out data.*

**H1-5.**  *A Random Forest model predicts anti-Vibrio activity with high out-of-sample performance, and predictor importance highlights Bacillus dominance and Vibrio presence as primary drivers.*

## 3. Results

### 3.1. Isolation, Morphotypic Screening, and In Vitro Antagonism of Native Probiotic Consortia

Cultivable bacteria were recovered from seawater collected from a larval reservoir during four consecutive months of *Penaeus vannamei* production, allowing the isolation of heterotrophic bacteria and presumptive *Bacillus* spp. under routine culture conditions. Viable growth was consistently obtained within the 10^−1^–10^−2^ dilution range for total heterotrophs and *Bacillus* spp., supporting the presence of an active cultivable fraction suitable for downstream functional screening.

**Figure 2:**
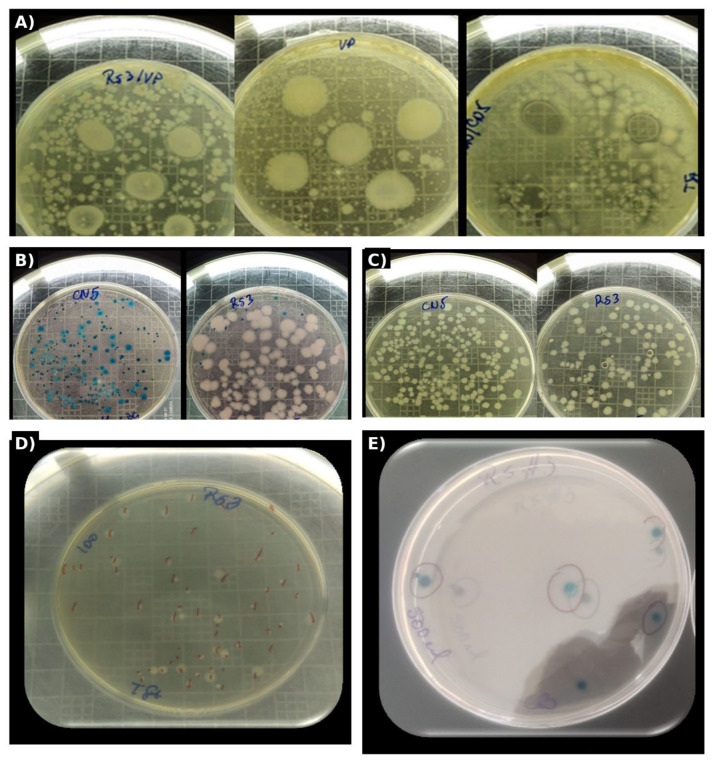
(**A–E**). Differential growth of CN5 and RS3 on TSA and Chromagar™ Bacillus and qualitative evidence of competitive exclusion against *V. parahaemolyticus* in simultaneous inhibition assays.

Differential growth on TSA and Chromagar™ *Bacillus* enabled a first discrimination between general heterotrophic morphotypes and spore-forming bacilli, respectively (Figure 2B–D).

From this pool, two native consortia were selected as probiotic candidates (CN5 and RS3), while pathogenic controls for functional assays were defined as *Vibrio parahaemolyticus* (V.p) and an invasive colony phenotype (C.INV), ensuring traceability across assays.

A preliminary morphotypic evaluation of colonies obtained from CN5 and RS3 showed round, creamy colonies with variable size, compatible with morphotypes of probiotic interest, and these isolates/consortia were preserved for subsequent molecular confirmation (16S rRNA).

Notably, Chromagar™ *Bacillus* plates revealed visually distinctive colony profiles (e.g., pigmented/blue-toned colonies vs. cream colonies), consistent with a bacilli-enriched community within the selected consortia (Figure 2B).

The antagonistic potential of the selected consortia was then evaluated using a simultaneous inhibition (competitive exclusion) approach (Figure 2A,E), where inhibition zones around inoculation points indicated suppression of pathogen growth in vitro.

In agreement with the study’s overall outcome, inhibition assays showed broad and consistent halos for RS3, CN5, and their combination, reaching ~99% antagonistic effectiveness against *V. parahaemolyticus* and supporting their candidacy as native biocontrol agents for sustainable aquaculture.

### 3.2. Treatment Effects and Temporal Dynamics of Antagonism Water Quality, and SEM-Aligned Indices

Across the four sampling points (Days 0, 10, 20, and 30), the physicochemical context remained broadly comparable among treatments. As shown in Table 6, dissolved oxygen, temperature, salinity, and pH followed similar temporal patterns across CTRL, CN5, RS3, and MIX, supporting the interpretation that between-group differences in downstream responses were not driven by major water-quality shifts. Consistent with this, the standardized water-quality z-index remained centered near zero over time, indicating limited physicochemical separation among experimental conditions.

In contrast, treatment-associated differences were evident in the biological response metrics. As summarized in Table 7, probiotic treatments (CN5, RS3, and MIX) showed consistently higher antagonistic activity (mean inhibition halo) relative to CTRL, alongside lower *Vibrio* relative abundance (“presence” index) and enriched *Bacillus* dominance. Microbial diversity indices also differed between probiotic conditions and the control, reinforcing that the primary separation among treatments was microbiological rather than physicochemical in nature.

**Figure 3:**
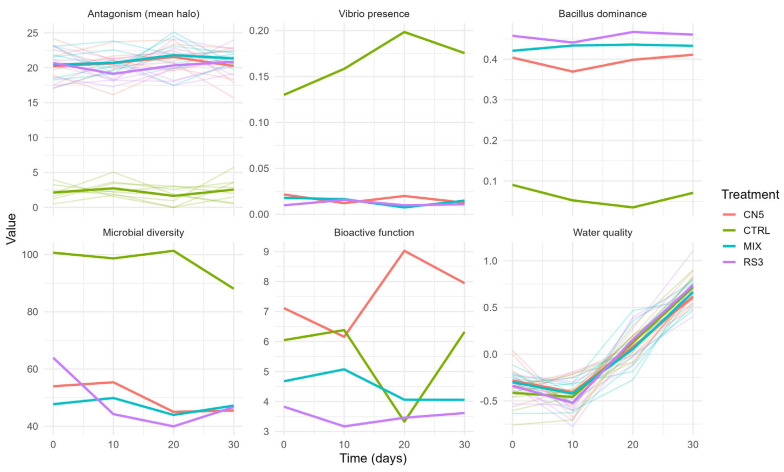
Temporal dynamics (replicates + treatment mean) of antagonism and SEM-aligned indices across time (0–30 days). Thin lines represent individual replicates and thick lines represent treatment means. Panels show: antagonism (mean inhibition halo), *Vibrio* presence, *Bacillus* dominance, microbial diversity, bioactive function, and water quality (z-index).

Temporal profiling (0, 10, 20, and 30 days) confirmed that probiotic-associated antagonism remained stable over time (≈20–22 mm), while the control remained low (≈0–6 mm), with clear separation of the control trajectories from probiotic treatments (Figure 3). Likewise, *Vibrio* presence remained consistently higher in CTRL, whereas *Bacillus* dominance remained consistently higher under CN5/MIX/RS3 (Figure 3), supporting treatment-driven stabilization of a *Bacillus*-dominant regime associated with antagonistic activity.

### 3.3. Species-Level Community Composition Across Treatments and Time

**Figure 4:**
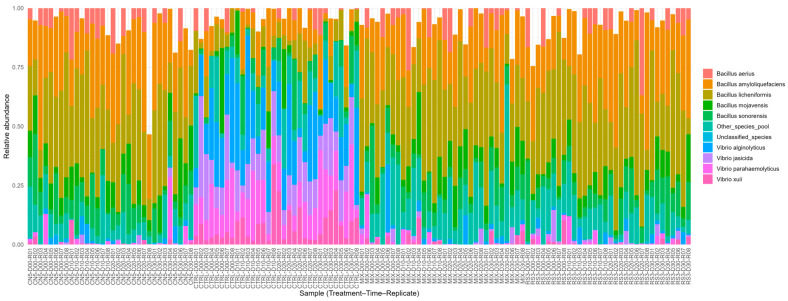
Species-level community composition across treatments and time (*n* = 128). Each bar represents one observation (Treatment–Time–Replicate). Colors denote relative abundances of detected species and aggregated categories (“Other_species_pool” and “Unclassified_species”).

**Figure 5:**
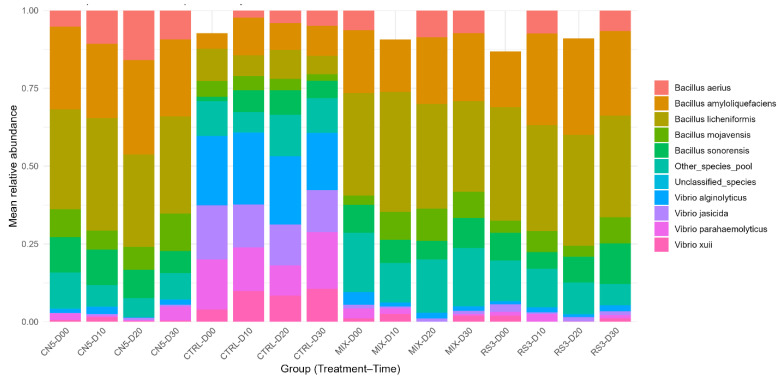
Mean species-level composition by treatment and time. Stacked bars represent mean relative abundances (*n* = 8 replicates per treatment–time group) for CTRL, CN5, MIX, and RS3 at 0, 10, 20, and 30 days.

16S rRNA amplicon-based profiling revealed a pronounced treatment-dependent restructuring of the microbial community (Figure 4 and Figure 5). When visualized at the level of individual observations (*n* = 128), stacked-bar profiles showed that CTRL samples were characterized by relatively higher contributions of *Vibrio* spp. (notably *V. alginolyticus*, *V. parahaemolyticus*, *V. jasicida*, and *V. xuii*), whereas probiotic treatments were consistently dominated by *Bacillus* spp., with recurrent prominence of *B. licheniformis* and *B. amyloliquefaciens* (Figure 4). Importantly, “Unclassified_species” was retained as an explicit component of the composition, ensuring transparency in species-level assignment.

To improve interpretability at the group level, mean composition was summarized for each treatment–time combination (16 groups; *n* = 8 replicates per group) (Figure 5). This aggregation confirmed that CTRL maintained a *Vibrio*-enriched profile throughout the time course, while CN5/MIX/RS3 maintained a stable *Bacillus*-enriched profile. Across probiotic treatments, *Bacillus* dominance was sustained over time, consistent with the functional phenotype of increased antagonism against *Vibrio* observed in vitro.

### 3.4. Multivariate Differentiation by PCA and Unsupervised k-Means Clustering

**Figure 6:**
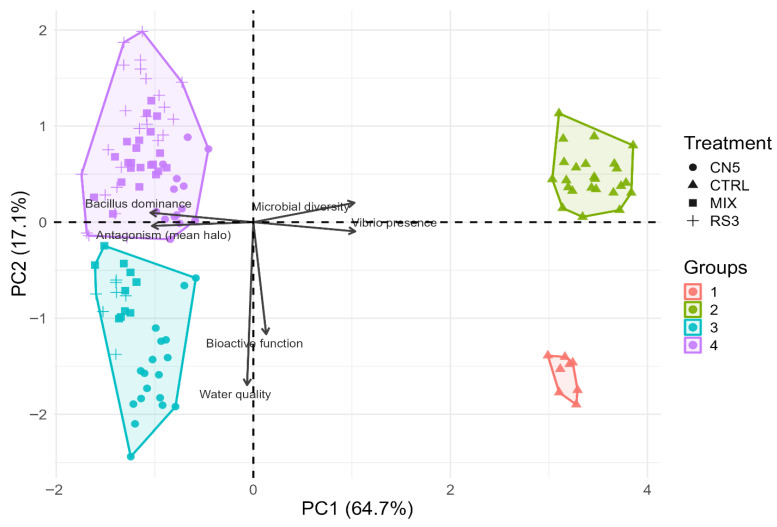
PCA biplot of SEM-aligned indices with k-means clustering (k = 4). Points represent observations; shapes denote treatment groups; convex hulls summarize cluster geometry. Arrows indicate variable loadings (direction and contribution) on PC1 and PC2. Dashed lines denote axes at zero.

PCA performed on standardized SEM-aligned indices (antagonism, *Vibrio* presence, *Bacillus* dominance, microbial diversity, bioactive function, and water quality) explained 81.8% of the total variance in the first two components (PC1 = 64.7%, PC2 = 17.1%) (Figure 6). PC1 primarily contrasted *Vibrio* presence and microbial diversity against *Bacillus* dominance and antagonism, providing a compact multivariate summary that is consistent with the species-level restructuring observed by amplicon-based taxonomic profiling (Figure 4 and Figure 5).

Unsupervised k-means clustering (k = 4) in PCA space supported robust group structure. CTRL samples were assigned to distinct clusters, whereas probiotic treatments concentrated into two major multivariate regimes (Table 8), consistent with probiotic-driven ecological states.

### 3.5. PLS-SEM Structural Model: Explained Variance, Effect Sizes, and Direct Effects

**Figure 7:**
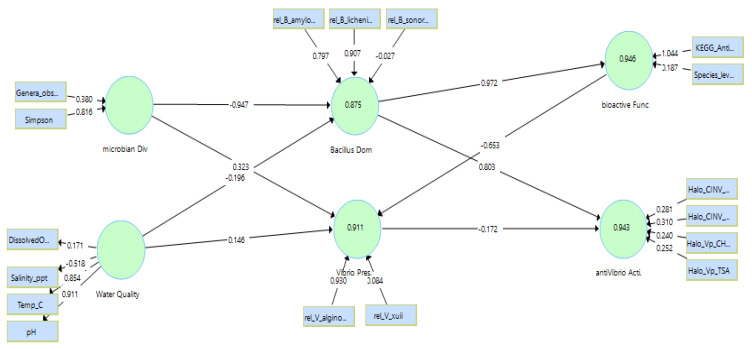
PLS-SEM structural model linking water quality, microbial diversity, *Bacillus* dominance, bioactive function, *Vibrio* presence, and anti-*Vibrio* activity. Values on endogenous constructs represent R^2^; values on arrows represent standardized path coefficients (β).

The PLS-SEM exhibited high explanatory power for endogenous constructs (Table 9), with R^2^ values of 0.875 (*Bacillus* dominance), 0.911 (*Vibrio* presence), 0.946 (bioactive function), and 0.943 (anti-*Vibrio* activity) (Figure 7). Effect-size assessment (f^2^) indicated very large contributions of microbial diversity to *Bacillus* dominance (f^2^ = 6.998) and of *Bacillus* dominance to bioactive function (f^2^ = 17.431), consistent with a cascade where community structure governs functional outputs and antagonism (Table 9).

Bootstrapping results (Table 10) indicated a strong positive effect of *Bacillus* dominance on anti-*Vibrio* activity (β = 0.803; t = 8.967; *p* < 0.001) and on bioactive function (β = 0.972; t = 188.864; *p* < 0.001). Microbial diversity strongly decreased *Bacillus* dominance (β = −0.947; t = 39.715; *p* < 0.001) and increased *Vibrio* presence (β = 0.323; t = 3.830; *p* < 0.001). Bioactive function showed a strong negative relationship with *Vibrio* presence (β = −0.653; t = 7.845; *p* < 0.001). Water-quality paths were not statistically supported at α = 0.05 (*p* ≥ 0.065), while the direct path from *Vibrio* presence to anti-*Vibrio* activity was marginal (β = −0.172; *p* = 0.057), suggesting that antagonism was primarily driven by the *Bacillus*-dominant state rather than by water chemistry alone.

### 3.6. Out-of-Sample Validation: Random Forest Prediction of Antagonism

**Figure 8:**
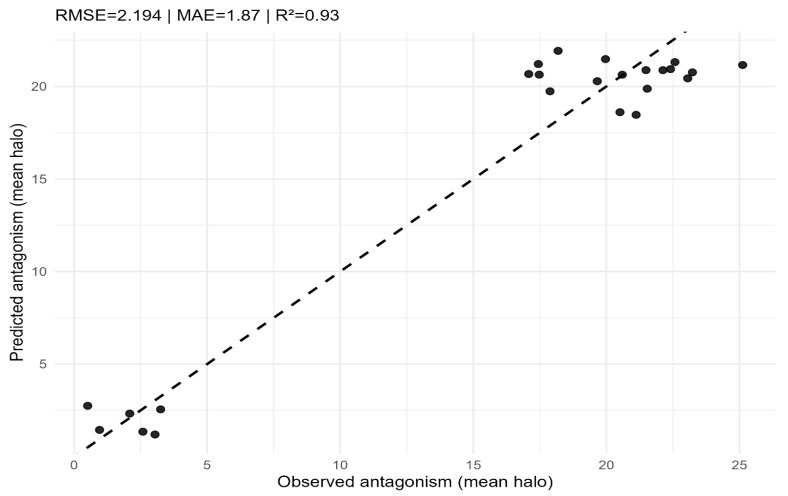
Random Forest regression: observed vs. predicted antagonism (mean halo) on the test set. The dashed line represents the 1:1 reference. Performance metrics (RMSE, MAE, R^2^) are reported in the panel.

**Figure 9:**
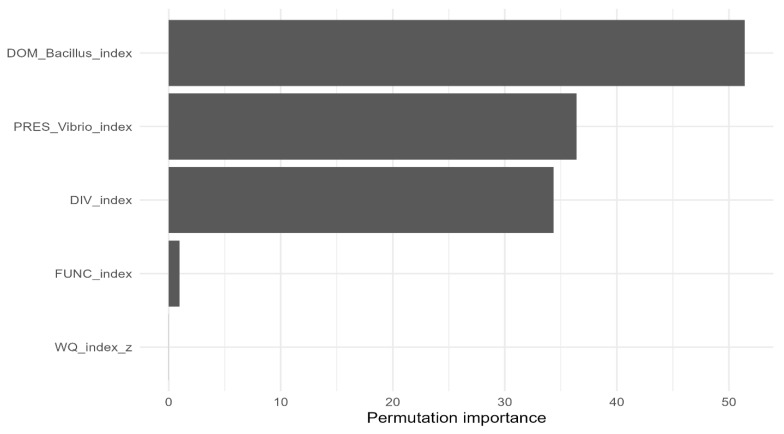
Random Forest permutation importance for antagonism prediction. Higher values indicate a stronger decrease in predictive performance when the corresponding predictor is permuted.

A Random Forest regressor trained on SEM-aligned indices achieved strong predictive performance on the held-out test set (RMSE = 2.194; MAE = 1.87; R^2^ = 0.93) (Figure 8). Permutation importance ranked *Bacillus* dominance as the most influential predictor of antagonism, followed by *Vibrio* presence and microbial diversity, whereas water quality contributed minimally (Figure 9). This ranking converges with the SEM results, reinforcing *Bacillus* dominance as the primary driver of the antagonistic phenotype.

### 3.7. Model Diagnostics: Global Fit and Collinearity

Global fit indices indicated SRMR = 0.149 for both saturated and estimated models, with discrepancy indices reported in Table 11. Collinearity diagnostics identified elevated VIF values for Temp_C (5.642) and pH (6.168), whereas most indicators exhibited acceptable collinearity levels (VIF ≈ 1–4) (Table 11). These diagnostics suggest redundancy in the water-quality block and should be considered when interpreting water-quality effects and when refining model specification.

## 4. Discussion

Across the 30-day hatchery cycle, probiotic consortia (CN5, RS3, MIX) produced consistently strong in vitro antagonism against *Vibrio parahaemolyticus* (Figure 2) and were associated with a shift of hatchery-water microbiomes toward *Bacillus*-dominant, *Vibrio*-depleted states (Figure 3, Figure 4 and Figure 5; Table 6 and Table 7), while physicochemical conditions remained broadly comparable among treatments. These patterns support an ecological framing of AHPND risk management in which prevention prioritizes community-level barriers rather than single-pathogen suppression [47,48].

### 4.1. Relevance of the Findings for AHPND-Risk Management in the Americas

Phylogenomic evidence supports that AHPND-associated *V. parahaemolyticus* lineages in Latin America may arise via multiple introduction pathways and/or local evolutionary trajectories, reinforcing the need for regionally validated prevention measures rather than assuming uniform strain ecology across continents [49]. Field-based detection work in the Americas further demonstrated that AHPND can become established in production landscapes where environmental and operational drivers (e.g., water exchange, organic loading, and temperature variability) create recurrent opportunities for toxigenic *Vibrio* expansion [50]. In this context, the high inhibitory performance observed for CN5/RS3/MIX in the competitive exclusion assay (Figure 2; inhibition halos clearly visible across plates) is not merely a laboratory phenotype—it represents a practical screening signal for identifying probiotic candidates capable of counteracting *V. parahaemolyticus* under conditions that often precede AHPND expression in farms.

A complementary concern is that AHPND systems may act as reservoirs for mobile genetic elements that carry not only virulence determinants but also antimicrobial resistance, which can be co-selected under farm pressures and complicate treatment options [51]. This strengthens the rationale for non-antibiotic strategies: our results support a pathway in which native consortia—selected from the same production environment—can generate strong in vitro suppression while also aligning with community patterns consistent with reduced *Vibrio* presence.

### 4.2. Water-Quality Context and “System State” Interpretation

Biofloc-based and related microbial-management approaches highlight that disease risk is often mediated by a system-level balance among C/N regime, microbial assimilation of nitrogenous wastes, and community competition, rather than by the pathogen alone [52]. Although this study is not framed as a full biofloc trial, the observed trajectories in the water-quality composite index across sampling moments can be interpreted as part of a broader “system maturation” process in which physicochemical stabilization may create a narrower niche window for opportunistic *Vibrio* proliferation. Importantly, the SEM structure (water quality → community structure → functional potential → anti-*Vibrio* activity) is consistent with the idea that environmental conditions shape microbial assembly, which then determines protective capacity.

### 4.3. Consortia Performance: From Inhibition Halos to Community Restructuring

Evidence from AHPND-related intervention studies shows that probiotic administration can improve survival and reshape bacterial communities when applied after or during pathogen pressure, but outcomes depend on strain ecology and environmental compatibility [53]. These findings (higher inhibition halos under probiotic treatments vs. control; Figure 2) align with the general expectation that multi-strain or community-based interventions can outperform single-mechanism approaches when competitive dynamics in the water column are central.

Consortium-based biocontrol has also been demonstrated in “hybrid” designs that combine microalgae and bacteria, where the protective effect emerges through both direct antagonism and resource/oxygen modulation [54]. Similarly, non-*Bacillus* probiotics (e.g., *Pseudoalteromonas*) have been reported to increase resistance of *P. vannamei* to AHPND-causing *Vibrio*, indicating that multiple taxonomic routes can reach a protective functional outcome [55]. In the present study, however, the sequencing-aligned indices suggest that protection is strongly coupled to *Bacillus* dominance, which is mechanistically plausible given this genus’ capacity for persistent colonization and metabolite production.

The MIX treatment conceptually resembles “synbiotic-like” logic reported for combined functional additives, where synergy between components can broaden antimicrobial and ecological effects [56]. Interpreted through the observed PCA separation and SEM paths, MIX can be discussed not only as “CN5 + RS3” but as a potential mechanism-complementation strategy, where distinct isolate pools contribute overlapping but not identical ecological functions.

### 4.4. AHPND as a Dysbiosis Trigger and the Meaning of Diversity Shifts

AHPND is increasingly recognized as a dysbiosis-associated process: disease states can correlate with destabilized community composition and functional signatures that favor opportunists, particularly *Vibrios*, under stressful or nutrient-rich conditions [57]. This is critical for interpreting the diversity results. If probiotic treatments reduce alpha-diversity while increasing *Bacillus* dominance, this does not necessarily indicate “worse” ecology; rather, it can represent a protective domination state where competitive exclusion constrains pathogen expansion (especially if water quality remains stable and antagonistic potential increases).

Experimental infection work further indicates that shrimp-associated microbiomes can respond differently to pathogenic vs. non-pathogenic *V. parahaemolyticus* exposures, supporting the idea that not all *Vibrio* signals are equal and that community response patterns can be diagnostic of risk states [58]. In larval contexts specifically, microbiome composition can shift markedly during disease, and community-based biomarkers have been proposed to distinguish healthy vs. compromised larval systems [59]. This design—combining culture-based antagonism with water-microbiome profiling—fits directly within this diagnostic-preventive paradigm.

Beyond *Bacillus*, actinomycete-based probiotics (e.g., *Streptomyces* formulations) have shown capacity to modulate shrimp gut microbiota and improve performance metrics, reinforcing that functional outcomes can be achieved through different microbial guilds and metabolite suites [60]. This supports a key interpretive point for the discussion: the central question is not whether the system becomes more diverse, but whether it becomes more functionally defensive and less permissive to *Vibrio* proliferation.

### 4.5. Mechanistic Plausibility: Bacillus-Driven Protection, Quorum Quenching, and Bioactive Metabolites

A closely comparable line of evidence shows that dietary supplementation with *Bacillus velezensis* can modulate shrimp microbiota and enhance resistance under AHPND-relevant contexts, supporting the plausibility of the *Bacillus*-dominance → protection pathway [61]. Mechanistically, quorum quenching and interference with *Vibrio* signaling has emerged as a credible route by which *Bacillus* strains reduce virulence expression and colonization efficiency, in addition to direct growth inhibition [62]. Inhibition of quorum-sensing-regulated behaviors (e.g., biofilm formation, motility, and coordinated virulence) is especially relevant because AHPND risk is linked not only to abundance but also to expression dynamics in the pathogen.

Recent evidence of quorum-sensing inhibition by *B. velezensis* against shrimp-pathogenic *Vibrio* spp. supports interpreting the observed inhibition halos as a composite of (i) antibacterial metabolites and (ii) anti-virulence interference that reduces competitive success [63]. This interpretation is consistent with the broader strategy of disrupting quorum sensing as a disease-control approach in aquaculture systems [64], and with the policy-level direction toward alternatives to antibiotics that reduce selection pressure for resistance while maintaining productivity [65]. Reviews focused on *Vibrios* specifically emphasize that quorum-sensing interference can target key behaviors central to pathogenic success, making it an attractive complement to community engineering [66].

At the metabolite level, *Bacillus* lipopeptides and biosurfactants are well-established as multifunctional compounds that can inhibit competitors and influence microbial surface interactions—mechanisms that align strongly with the visible inhibition halos and with a SEM pathway linking *Bacillus* dominance to inferred bioactive functional potential [67]. In the discussion, this supports framing *Bacillus* dominance not as a taxonomic endpoint, but as a functional driver capable of reshaping both competitive outcomes and predicted pathway signatures.

### 4.6. Interpreting Inferred Functions Responsibly

Because the “bioactive function index” is inferred from amplicon profiles, it should be discussed with appropriate caution. Predictive functional profiling (e.g., PICRUSt-style inference) can generate useful hypotheses about pathway shifts when shotgun metagenomics is not available, and it has been widely used to bridge composition-to-function narratives [68]. However, functional redundancy and taxonomic-function decoupling can limit the resolution of inference: distinct communities may encode overlapping functional capacities, and function can vary at strain level within a genus [69]. Therefore, the discussion should position inferred functions as directional evidence consistent with antagonism phenotypes, not as definitive proof of metabolite production.

A second interpretive safeguard is the compositional nature of relative abundance data: changes in one taxon necessarily affect the proportional representation of others, which can amplify apparent shifts unless interpreted using appropriate normalization and modeling logic [70]. This is relevant for explaining why *Bacillus* dominance may coincide with decreased alpha-diversity and reduced relative abundance of other groups, without implying absolute elimination.

### 4.7. Integrated Analytics: PCA + Random Forest + PLS-SEM as Convergent Evidence

The multivariate results can be framed as convergent evidence that treatments correspond to distinct ecological regimes. Random forests provide a robust, nonlinear framework for classification/regression that can capture interactions among predictors—important in microbiome datasets where effects are rarely purely additive [71]. In microbial ecology, random forest approaches have been successfully used to identify discriminant taxa or features and to support predictive interpretation of community shifts [72]. At the same time, best-practice frameworks emphasize that microbiome machine learning must be interpreted carefully (avoiding leakage, ensuring cross-validation discipline, and treating feature importance as suggestive rather than causal) [73]. In the present study, Random Forest can be positioned as a confirmatory ranking tool that highlights which SEM-aligned indices (e.g., *Bacillus* dominance, *Vibrio* presence, water quality, diversity) most strongly discriminate treatments/time points.

Because implementation details can influence reproducibility and performance, noting the use of a well-established random forest implementation for high-dimensional data is defensible this context [74]. The central narrative then becomes:

- PCA: demonstrates separation of treatment regimes in reduced dimensional space (ecological-state visualization).
- Random Forest: ranks which indices best predict regime membership (predictive corroboration).
- PLS-SEM: tests an explicit directed mechanism (pathway-based explanation).

Variance-based SEM interpretation is strengthened when estimation logic, reliability, and predictive relevance are reported transparently, consistent with guidance for PLS-oriented models [75,76]. In this study, combining SEM-aligned paths with out-of-sample validation supports a prediction-oriented interpretation of microbiome management mechanisms while maintaining appropriate caution about causal claims.

### 4.8. Limitations and Implications for Application

Two limitations should be stated clearly and framed constructively. First, functional inference should be treated as hypothesis-generating and ideally validated in follow-up work using targeted metabolomics (e.g., lipopeptides), qPCR of functional genes, or shotgun metagenomics, consistent with recognized limits of inference and redundancy [68,69]. Second, the inhibition-halo assay is a controlled approximation of competitive outcomes; translating effects to operational settings requires validation under farm-like complexity and dosing regimes, consistent with the broader experience that probiotic efficacy is context-dependent [48,53].

## 5. Conclusions

This study demonstrates that native *Bacillus*-based probiotic consortia (CN5, RS3, and their combination) are consistently associated with strong in vitro antagonistic activity against *Vibrio parahaemolyticus* and with pronounced restructuring of shrimp hatchery water microbiomes toward *Bacillus*-dominated states. By integrating culture-based inhibition assays with amplicon sequencing, functional inference, and multivariate and structural modeling, the results support a coherent ecological pattern in which *Bacillus* dominance and reduced *Vibrio* presence co-occur with increased antagonistic potential. Importantly, the findings do not establish direct causality between specific metabolites, gene products, or in vivo disease outcomes, nor do they demonstrate protection against AHPND under farm or challenge conditions. Functional predictions were inferred from 16S rRNA gene data and therefore represent potential rather than experimentally confirmed metabolic activity. Water-quality effects were limited within the modeled system and should be interpreted cautiously. Consequently, while the data support the suitability of native probiotic consortia as promising biocontrol candidates and as drivers of protective microbial community states, further validation under controlled infection trials and commercial-scale conditions is required. Overall, this work contributes mechanistic and ecological evidence that community-level microbial management, rather than single-pathogen targeting, is a viable framework for improving disease resilience in shrimp hatchery environments.

## Figures and Tables

**Table 1:** Experimental design and sampling structure (*n* = 128).

Factor	Levels	Details
Time	4	0, 10, 20, 30 days (10-day intervals)
Treatment	4	CN5, RS3, MIX (CN5 + RS3), CTRL
Replication	8	Independent biological replicates per treatment–time
Total observations	128	4 × 4 × 8

**Table 2:** Evaluated variables, measurement, and analytical mapping.

Domain (SEM-Aligned)	Operational Definition	Data Source	Notes/Related Refs.
Anti-*Vibrio* activity	Mean inhibition halo (mm)	Culture assay	In vitro antimicrobial evaluation [22]
*Vibrio* presence	Relative abundance (RA) of *Vibrio* spp. $(n_{Vibrio}$$/N_{total}$).	16S taxonomic table	*Vibrio* ecology & identification [10,11]
*Bacillus* dominance	Relative abundance (RA) of *Bacillus* spp. $(n_{Bacillus}$$/N_{total}$)	16S taxonomic table	*Bacillus* probiotics in shrimp [17,18,19]
Microbial diversity	Observed genera + Simpson (index)	ASV table	Alpha diversity via phyloseq workflow [30]
Bioactive function	Aggregated inferred pathways (e.g., antimicrobial-related)	PICRUSt2 + KEGG + eggNOG/COG	Functional inference [32,33,34,35]
Water quality	z-index of DO, salinity, temperature, pH	Field/lab measures	Modeled as latent construct in SEM

**Table 3:** Culture-based isolation and screening workflow.

Step	Medium/Method	Purpose	Output
Serial dilution	10^−1^–10^−2^ in sterile saline	Reduce density; isolate colonies	Dilution series
Plating	TSA; Chromagar™ *Bacillus*	General heterotrophs vs. *Bacillus* enrichment	Mixed vs. *Bacillus*-like morphotypes
Morphotyping	Colony traits	Select distinct candidates	Candidate isolates
Purification & storage	Re-streak; −80 °C stocks	Preserve strains/consortia	CN5, RS3 isolate pools

**Table 4:** Sequencing and bioinformatics pipeline (reproducibility map).

Stage	Tool/Database	Key Operation	Ref.
Denoising & ASVs	DADA2 (via QIIME2)	Error-correction, ASV inference	[23,24]
Trimming	Cutadapt; Trimmomatic	Adapter removal; quality trimming	[25,26]
Auxiliary ops	VSEARCH	Dereplication/support operations	[27]
Taxonomy	SILVA v138	Classifier training & assignment	[28]
R integration	phyloseq	Alpha diversity; composition	[30]
Differential abundance	DESeq2	Count-model testing	[31]

**Table 5:** Statistical and modeling plan (what was used vs. not used).

Analysis Objective	Method	Output
Assay comparisons	Anderson–Darling; Levene; one-way ANOVA; Tukey (α = 0.05)	Group differences
Community composition	Relative abundance summaries	Taxa profiles
Differential abundance	DESeq2	log2FC + adjusted *p*
Beta-diversity	UniFrac	Distance matrix
Ordination	PCA (FactoMineR)	PC scores/loadings
Unsupervised regimes	k-means in PC space	Cluster membership
Causal/latent modeling	PLS-SEM	β paths, R^2^, validity
Predictive validation	Random Forest regression	RMSE/MAE/R^2^ + importance

**Table 6:** Water-quality parameters (mean ± SD) measured in situ across treatments and sampling days (0, 10, 20, 30).

Parameter	Day	CTRL	CN5	RS3	MIX
**DO (mg L^−1^)**	0	6.12 ± 0.25	6.13 ± 0.22	6.11 ± 0.27	6.14 ± 0.26
	10	4.93 ± 0.42	4.98 ± 0.23	5.01 ± 0.21	4.96 ± 0.29
	20	5.05 ± 0.23	5.08 ± 0.32	5.10 ± 0.23	5.06 ± 0.19
	30	5.92 ± 0.14	5.90 ± 0.22	6.10 ± 0.15	5.95 ± 0.18
**Temperature (°C)**	0	28.05 ± 0.14	28.06 ± 0.24	27.99 ± 0.12	28.00 ± 0.18
	10	28.24 ± 0.12	28.23 ± 0.24	28.07 ± 0.16	28.31 ± 0.20
	20	29.12 ± 0.22	29.18 ± 0.25	29.06 ± 0.16	29.14 ± 0.22
	30	29.59 ± 0.28	29.40 ± 0.12	29.60 ± 0.13	29.57 ± 0.10
**Salinity (ppt)**	0	31.62 ± 0.14	31.65 ± 0.12	31.62 ± 0.18	31.63 ± 0.16
	10	31.79 ± 0.11	31.77 ± 0.12	31.77 ± 0.11	31.79 ± 0.12
	20	33.67 ± 0.21	33.73 ± 0.20	33.68 ± 0.19	33.73 ± 0.18
	30	33.75 ± 0.16	33.79 ± 0.15	33.78 ± 0.15	33.78 ± 0.15
**pH**	0	8.13 ± 0.05	8.12 ± 0.04	8.12 ± 0.05	8.13 ± 0.05
	10	8.14 ± 0.04	8.13 ± 0.04	8.13 ± 0.04	8.13 ± 0.04
	20	8.17 ± 0.04	8.17 ± 0.04	8.15 ± 0.04	8.16 ± 0.04
	30	8.16 ± 0.04	8.17 ± 0.03	8.17 ± 0.04	8.16 ± 0.04

**Table 7:** Treatment-level summary of key variables (mean ± SD; *n* = 32 per treatment).

Variable	CN5	CTRL	MIX	RS3
Antagonism (mean halo, mm)	20.673 ± 2.208	2.266 ± 1.347	21.068 ± 1.930	20.254 ± 1.990
*Vibrio* presence (index)	0.0166 ± 0.0043	0.1656 ± 0.0254	0.0142 ± 0.0041	0.0116 ± 0.0025
*Bacillus* dominance (index)	0.3957 ± 0.0161	0.0622 ± 0.0210	0.4308 ± 0.0060	0.4565 ± 0.0097
Microbial diversity (index)	49.974 ± 4.822	97.182 ± 5.450	47.194 ± 2.157	48.720 ± 9.282
Bioactive function (index)	7.561 ± 1.075	5.519 ± 1.291	4.468 ± 0.439	3.518 ± 0.244
Water quality (z-index)	0.001 ± 0.432	−0.008 ± 0.504	−0.001 ± 0.462	0.008 ± 0.524

**Table 8:** k-Means cluster membership by treatment (counts and within-treatment %; k = 4).

Treatment	Cluster 1	Cluster 2	Cluster 3	Cluster 4
CN5 (*n* = 32)	0 (0.0%)	18 (56.2%)	14 (43.8%)	0 (0.0%)
CTRL (*n* = 32)	24 (75.0%)	0 (0.0%)	0 (0.0%)	8 (25.0%)
MIX (*n* = 32)	0 (0.0%)	10 (31.2%)	22 (68.8%)	0 (0.0%)
RS3 (*n* = 32)	0 (0.0%)	7 (21.9%)	25 (78.1%)	0 (0.0%)

**Table 9:** Structural model quality: explained variance (R^2^, adjusted R^2^), effect sizes (f^2^), and predictive relevance (Q^2^_predict).

Endogenous Construct	R^2^	Adjusted R^2^	Key f^2^ Contributors (Toward the Endogenous Construct)	RMSE	MAE	Q^2^_Predict
*Bacillus* dominance	0.875	0.873	Microbial diversity → *Bacillus* (6.998); Water quality → *Bacillus* (0.300)	0.383	0.288	0.858
*Vibrio* presence	0.911	0.909	Microbial diversity → *Vibrio* (0.225); Water quality → *Vibrio* (0.208)	0.453	0.365	0.801
Anti-*Vibrio* activity	0.943	0.942	*Bacillus* → Activity (0.563); *Vibrio* → Activity (0.026)	0.404	0.329	0.841
Bioactive function	0.946	0.945	*Bacillus* → Function (17.431); *Vibrio* → Function (0.947)	0.470	0.394	0.785

**Table 10:** Bootstrapping results for direct effects in the structural model.

Path	β (Original Sample)	Mean (Bootstrap)	STDEV	t	*p*
*Bacillus* dominance → Anti-*Vibrio* activity	0.803	0.825	0.090	8.967	<0.001
*Bacillus* dominance → Bioactive function	0.972	0.972	0.005	188.864	<0.001
*Vibrio* presence → Anti-*Vibrio* activity	−0.172	−0.149	0.090	1.901	0.057
Water quality → *Bacillus* dominance	−0.196	−0.122	0.106	1.842	0.065
Water quality → *Vibrio* presence	0.146	0.113	0.096	1.521	0.128
Bioactive function → *Vibrio* presence	−0.653	−0.689	0.083	7.845	<0.001
Microbial diversity → *Bacillus* dominance	−0.947	−0.934	0.024	39.715	<0.001
Microbial diversity → *Vibrio* presence	0.323	0.287	0.084	3.830	<0.001

**Table 11:** Global fit and collinearity diagnostics (VIF).

(**A**) **Global fit indices**			
**Index**	Saturated model	Estimated model	
SRMR	0.149	0.149	
$D_{ULS}$	3.381	3.394	
$D_{G}$	2.682	3.049	
χ2	1057.135	1157.327	
NFI	0.681	0.651	
(**B**) **Variance inflation factors (VIF) for indicators**			
**Indicator**	VIF	Indicator	VIF
DissolvedO2_mgL	1.394	Salinity_ppt	1.273
Genera_observed	1.103	Simpson	1.103
Halo_CINV_CHROM	3.893	Species_level_%	1.116
Halo_CINV_TSA	4.206	Temp_C	5.642
Halo_Vp_CHROM	3.760	pH	6.168
Halo_Vp_TSA	3.961	rel_B_amyloliquefaciens	1.149
KEGG_AntimicrobialScore_meta	1.116	rel_B_licheniformis	1.349
rel_B_sonorensis	1.319	rel_V_alginolyticus	3.217
rel_V_xuii	3.217		

## Data Availability

The original contributions presented in this study are included in the article. Further inquiries can be directed to the corresponding author.
